# Record-high thermal barrier of the relaxation of magnetization in the nitride clusterfullerene Dy_2_ScN@C_80_-*I_h_*†

**DOI:** 10.1039/c7cc03580b

**Published:** 2017-07-11

**Authors:** D. S. Krylov, F. Liu, S. M. Avdoshenko, L. Spree, B. Weise, A. Waske, A. U. B. Wolter, B. Büchner, A. A. Popov

**Affiliations:** Leibniz Institute for Solid State and Materials Research, 01069 Dresden, Germany

## Abstract

The Dy-Sc nitride clusterfullerene Dy_2_ScN@C_80_-*I_h_* exhibits slow relaxation of magnetization up to 76 K. Above 60 K, thermally-activated relaxation proceeds *via* the fifth-excited Kramers doublet with the energy of 1735 ± 21 K, which is the highest barrier ever reported for dinuclear lanthanide single molecule magnets.

The encapsulation of metal clusters within the fullerene cage has multiple structural and physical consequences for the properties of endohedral metallofullerenes (EMFs).^1^ In particular, unusual magnetic properties can be achieved in EMFs when encapsulated metals are lanthanides. The interaction of lanthanide ions with negatively-charged non-metal ions (nitride, carbide, oxide) within the endohedral cluster leads to a large magnetic anisotropy of the former,^2^ which is crucial for the single molecule magnetism (SMMs are the molecules with bistable magnetic ground state showing slow relaxation of the magnetization).^3^,^4^

DySc_2_N@C_80_-*I_h_* was the first EMF to show SMM properties.^5^ In later reports, Dy_2_ScN@C_80_-*I_h_*^6^ as well as isostructural Dy_2_TiC@C_80_^7^ were found to exhibit SMM behaviour. A slow relaxation of magnetization was also reported in nitride and carbonitride clusterfullerenes with non-Kramers Ho and Tb ions,^8^ but with much shorter relaxation times than in Dy-EMFs.

The high magnetic anisotropy of Dy ions in EMFs is expected to lead to large barriers of the thermally-activated relaxation of magnetization *via* the Orbach relaxation mechanism. Theoretical predictions for such barriers exceed 1000 K and approach 2000 K.2a,d However, such barriers have not been observed experimentally yet, which may be caused by the limited temperature ranges, in which magnetic properties of EMFs were studied. For Dy_2_ScN@C_80_, which is the best EMF-SMM reported so far, detailed magnetic studies have been limited to temperatures below 20 K.^6^ They revealed an Orbach relaxation mechanism with the barrier of 8.5 K assigned to the exchange/dipolar excited state, in which Dy ions are coupled antiferromagnetically. In this work we use ac magnetometry to unravel the mechanism of the relaxation of magnetization in Dy_2_ScN@C_80_ up to 76 K and find that the compound has an unprecedentedly high relaxation barrier exceeding all reported values for polynuclear systems and approaching the highest value of 1815(1) K reported for the single-ion Dy-SMM.4c

Dy_2_ScN@C_80_-*I_h_* has been synthesized by arc-discharge synthesis as described previously.^9^ In brief, a mixture of Dy_2_O_3_, Sc_2_O_3_, and guanidine thiocyanate was mixed with graphite powder and packed into the drilled-out graphite rods, which were then evaporated in 180 mbar He atmosphere. EMFs were Soxhlet-extracted from the resulting soot by CS_2_ and separated by HPLC (see ESI† for further details).

In the previous work, the molecular structure of Dy_2_ScN@C_80_ was established by spectroscopic techniques.9b Here we report on the crystallographic elucidation of its molecular structure. A cocrystal Dy_2_ScN@C_80_-*I_h_*⋅Ni^II^(OEP)⋅2C_6_H_6_ (OEP = octaethyl-porphyrin) was obtained by layering a benzene solution of Ni^II^(OEP) over the solution of Dy_2_ScN@C_80_-*I_h_* in carbon disulphide.§^10^ Fig. 1a displays the relative orientation of the fullerene and Ni^II^(OEP) molecules in the crystal. The nearest cage-Ni contact (between Ni1P and C1A) is determined to be 2.793(7) Å. Thanks to the coordination to the bowl-shaped Ni^II^(OEP), the C_80_-*I_h_*(7) cage is fully ordered, whereas the Dy_2_ScN cluster is disordered between two sites with fractional occupancies of 0.69 and 0.31. DFT calculations for Y_2_ScN@C_80_-*I_h_* (see ESI†) show that both sites correspond to energy minima with the energy difference of 6.3 kJ mol^−1^ in favour of the more abundant configuration. In both sites, Sc and one of the Dy ions are directed towards Ni^II^(OEP), whereas the second Dy is facing the opposite side of the cage. A similar arrangement of the cluster was reported in the Gd_2_ScN@C_80_-*I_h_* Ni^II^(OEP)⋅2C_6_H_6_ crystal.^11^ Furthermore, this arrangement follows the general pattern observed for M_3_N@C_80_-*I_h_*^12^ and M_2_TiC@C_80_-*I_h_*^13^ cluster-fullerenes co-crystallized with Ni^II^(OEP). Since only the *I_h_* isomer is considered in this work, in the following we will omit the designation of the fullerene cage.

The magnetic properties of a Dy_2_ScN@C_80_ powder sample were studied by SQUID magnetometry. Fig. 2 shows the magnetization curves measured at low temperatures. When the magnetic field is swept with a rate of 2.9 mT s^−1^, the hysteresis of the magnetization is observed between 1.8 and 7 K (Fig. 2; the coercive field at 2 K is 0.7 T). The blocking temperature of magnetization, *T*_B_, is defined as the position of the peak on the *χ*–*T* curve of a zero-field-cooled sample (*χ* is the magnetic susceptibility). For Dy_2_ScN@C_80_, *T*_B_ depends on the temperature sweep rate and varies between 7 and 8 K when the rate is increased from 2 K min^−1^ to 5 K min^−1^ (Fig. 2, inset).

The magnetization relaxation times below 5 K can be determined from the decay of the magnetization measured with dc-magnetometry. Unfortunately, determination of the relaxation time in such measurements is rather ambiguous as relaxation curves at low temperature usually show a form of multiexponential decay. In a previous work, a bi-exponential fitting was used and the longer times were interpreted as intrinsic to the SMM.^6^ Here we use a stretched exponential fitting to obtain average relaxation times. The obtained relaxation times follow an Arrhenius behaviour corresponding to the Orbach relaxation process:

(1) $$\tau^{-1}=\tau_{0}^{-1}\text{exp}(-U_{\text{eff}}/T)$$

where *U*_eff_ is the effective barrier and *τ*_0_ is the attempt time. Fitting the dc data with eqn (1) (Fig. 3) gives an energy barrier of 10.7 ± 0.3 K and an attempt time of *τ*_0_ = 11.9 ± 1.5 s. In ref. 6, the use of the bi-exponential fit for the magnetization decay curves resulted in a barrier of 8.5 ± 0.5 K and much longer *τ*_0_ of 56.5 ± 9.8 s. The *U*_eff_ value of 10.7 K (8.5 K in ref. 6) corresponds to the exchange/dipolar barrier: In the ground state, magnetic moments of Dy ions are coupled ferromagnetically, whereas flipping of the spin on one of the Dy centres gives an antiferromagnetically coupled state with the energy *U*_eff_.^6^ Dipolar interaction contributes 4.6 K, *i.e.* roughly a half of the barrier (see ESI†).

Above 5 K, the rates of the relaxation of magnetization were studied with ac magnetometry. In these measurements, we used two Quantum Design magnetometers, MPMS XL (with a reliable sensitivity from 0.1 Hz to *ca.* 500 Hz) and a PPMS system (frequency range 10 Hz–10 kHz, but with a rather poor sensitivity below 100 Hz). In the temperature range of 12–45 K, the measurements revealed distorted *χ*″ peaks indicating that the relaxation of magnetization proceeds *via* two channels with distinct characteristic times (Fig. 4). The data were then fitted using either one or two relaxation times (discussed hereafter as single-τ and double-τ models, respectively; see inset in Fig. 4a). The single-τ model gives an average time of the two relaxation processes. Both short-τ and long-τ processes are temperature dependent. Interestingly, the long-τ relaxation channel dominates at lower temperatures, whereas an increase of the statistical weight of the short-τ relaxation channel is observed at higher temperatures (Fig. 3). Note that the local coordination sphere of the two Dy ions in the Dy_2_ScN@C_80_ molecule is slightly different, and hence the coexistence of the two concomitant relaxation processes may be caused by a different relaxation behaviour of Dy centres in one molecule. The coexistence of at least two relaxation channels is often observed in di- and polynuclear SMMs with non-equivalent lanthanide centres.3b,^14^ The nature of the relaxation mechanisms for these processes is not clear yet. Traditionally, sub-barrier relaxation in SMMs is ascribed to the Raman mechanism, but the recent analysis of spin-phonon coupling and dynamics by Lunghi *et al.* suggested that low-frequency unharmonic phonons with finite linewidth may cause Arrhenius-like behaviour at low temperatures.^15^

Above 45 K, the two relaxation processes cannot be distinguished anymore, and the single-τ behaviour is observed up to 76 K (above this temperature, the peak in *χ*″ is beyond the accessible frequency range). Between 63 and 76 K, the data points show an Arrhenius behaviour (Fig. 3) with the effective energy barrier of 1735 ± 21 K and an attempt time *τ*_0_ = 2.39 x 10^−15^ s. Thus, Dy_2_ScN@C_80_ has one of the highest magnetization relaxation barriers ever reported for SMMs and is second to only [Dy(O^*t*^Bu)_2_(py)_5_][BPh_4_] with the energy barrier of 1815(1) K.4c Before this work, the highest thermal relaxation barriers among dinuclear lanthanide systems were found in isocarbonyl-ligated Dy-metallocene [Cp*_2_Dy-{μ-(OC)_2_FeCp}]_2_ (953 K)^16^ and the hydroxide-bridged five-coordinate Dy^III^ dimer (721 K).^17^

In weakly-coupled dinuclear SMMs, the relaxation of magnetization is believed to proceed *via* single-ion states.14d,^18^ To clarify the relaxation mechanism and the nature of the observed barrier in Dy_2_ScN@C_80_, we performed *ab initio* computations. As the apparent geometry of the Dy_2_ScN cluster in the crystal is distorted by the disorder, we used coordinates from the X-ray structure only as a starting point for the DFT geometry optimization of the Y_2_ScN@C_80_ molecule.¶ One of the Y ions in the optimized structure was then replaced by Dy, and single-point CASSCF/RASSI/ANO-RCC-VDZ calculations were performed using the Molcas 8.0 code.^19^ The crystal-field (CF) parameters derived by the SINGLE-ANISO module^20^ were then used in the PHI code^21^ for further model Hamiltonian calculations. Fig. 5 shows the CF energy levels of Dy1 and Dy2 as well as transition probabilities between the CF states. Similar to the earlier studies, 2a our calculations predict a large CF splitting reaching 1400 cm^−1^ for both Dy ions. The CF is highly axial and gives 8 Kramers doublets (KDs) with the ground state corresponding to *J*_z_ = ±15/2. Several higher energy KDs also have almost pure *m*_J_ composition. As a result, transition probabilities within the KDs are very low up to the fourth CF excited state. At higher energies, *m*_J_ states mix significantly, leading to the dramatic increase of the transition probabilities (Fig. 5). Based on these calculations, the relaxation of the magnetization in Dy_2_ScN@C_80_ is expected to proceed efficiently *via* the fifth KDs of individual Dy centres. The computed energies of these states (1618 K for Dy1 and 1641 K for Dy2) are only slightly lower than the experimental value of 1735 K. The deviation is likely to be due to the insufficient accuracy of the CASSCF model (such as its lack of dynamic correlation), but may be also caused by other relaxation channels (*e.g.*, the relaxation *via* the sixth or higher CF states).

To conclude, this Communication reports on the studies of the dynamic magnetic properties of Dy_2_ScN@C_80_ and reveals that it has a very high relaxation barrier of magnetization, 1735 ± 21 K. Based on *ab initio* calculations, the barrier is assigned to a thermally-activated relaxation *via* the fifth crystal-field excited state of individual Dy centres. High axiality of the CF states is crucial for reaching high barriers of the relaxation of magnetization as it prevents the relaxation *via* low-energy KDs.^22^ In Dy_2_ScN@C_80_, despite the low symmetry of the Dy coordination sphere, the high axiality is achieved because of the short distance between Dy and the nitride ion. Further increase of the barrier in Dy–nitride clusterfullerenes may be thus achieved by geometrically forcing the Dy–N distance to be shorter by either substituting Sc by a larger diamagnetic ion, or by considering a smaller carbon cage.

## Supplementary Material

† Electronic supplementary information (ESI) available: Additional experimental details, HPLC separation, magnetic measurement. CCDC 1547067. For ESI and crystallographic data in CIF or other electronic format see DOI: 10.1039/c7cc03580b

ESI

## Figures and Tables

**Fig. 1:**
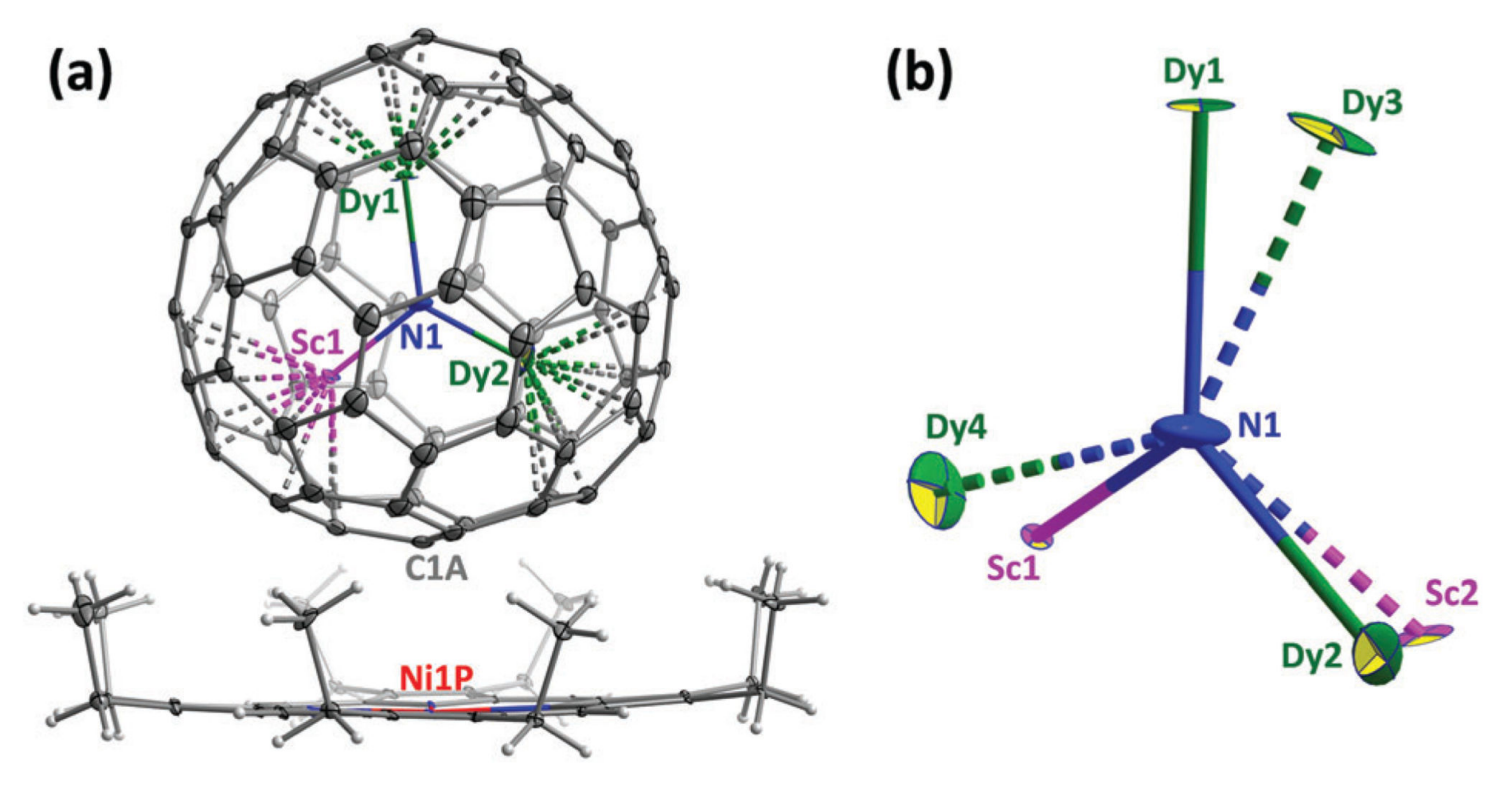
(a) Single-crystal X-ray structure of Dy_2_ScN@C_80_-*I_h_*(7)⋅Ni^II^(OEP)·2C_6_H_6_. Solvent molecules are omitted for clarity, only the main site of the cluster is shown (fractional occupancy 0.69). (b) Two sites of the Dy_2_ScN cluster in the structure, the main site (Dy1, Dy2, and Sc1) is shown with solid bonds to N1, minor site (Dy3, Dy4, and Sc2) with dash-line bonds to N1, only one nitrogen site N1 is refined. All displacement parameters are shown at the 30% probability level.

**Fig. 2:**
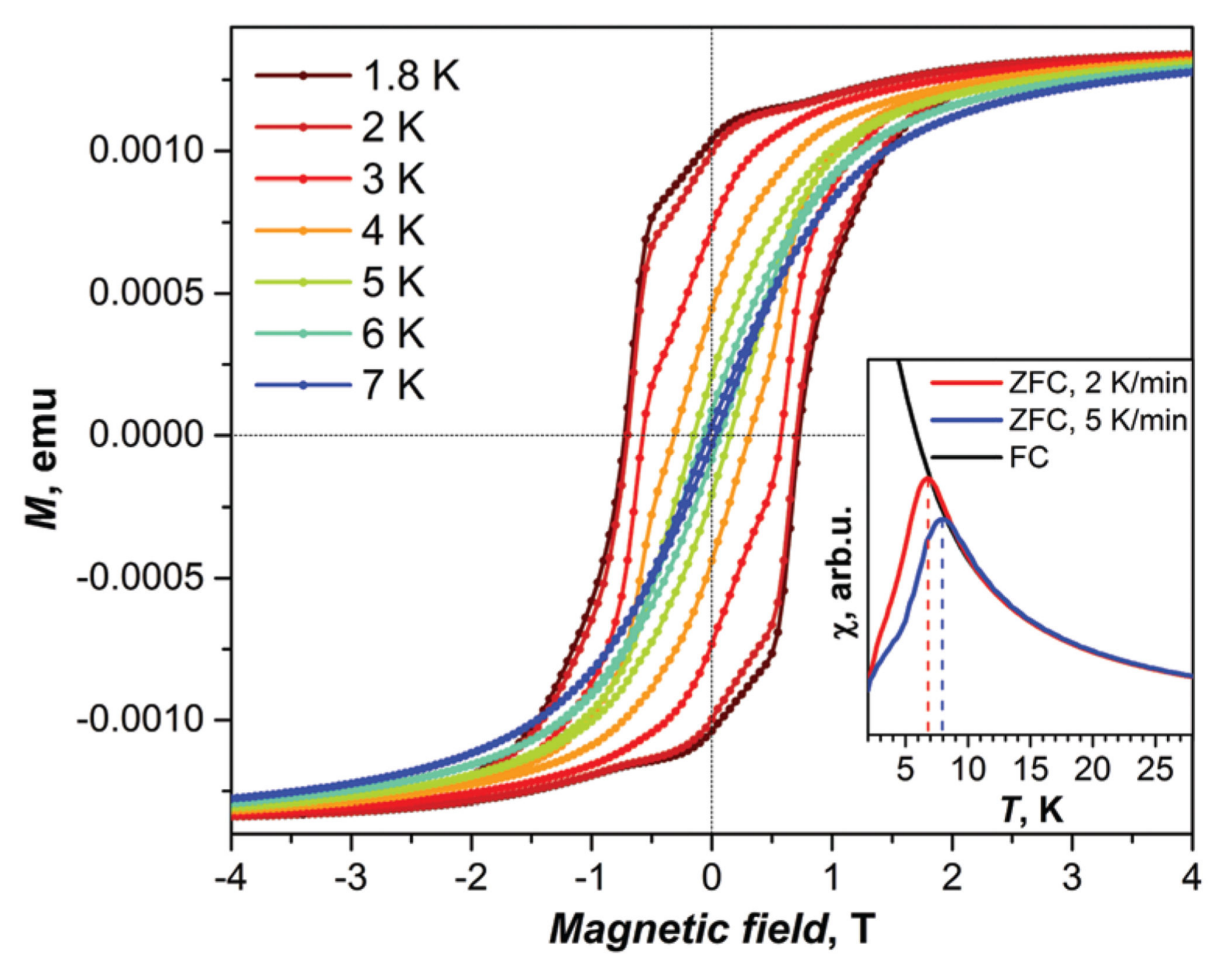
Magnetization curves of Dy_2_ScN@C_80_ measured at 1.8–7 K (sweep rate of 2.9 mT s^−1^). At 8 K (not shown) the hysteresis is closed. The inset shows the blocking temperature of magnetization (*T*_B_) as the peak in the susceptibility of zero-field cooled (ZFC) sample as opposed to the field-cooled (FC) sample.

**Fig. 3:**
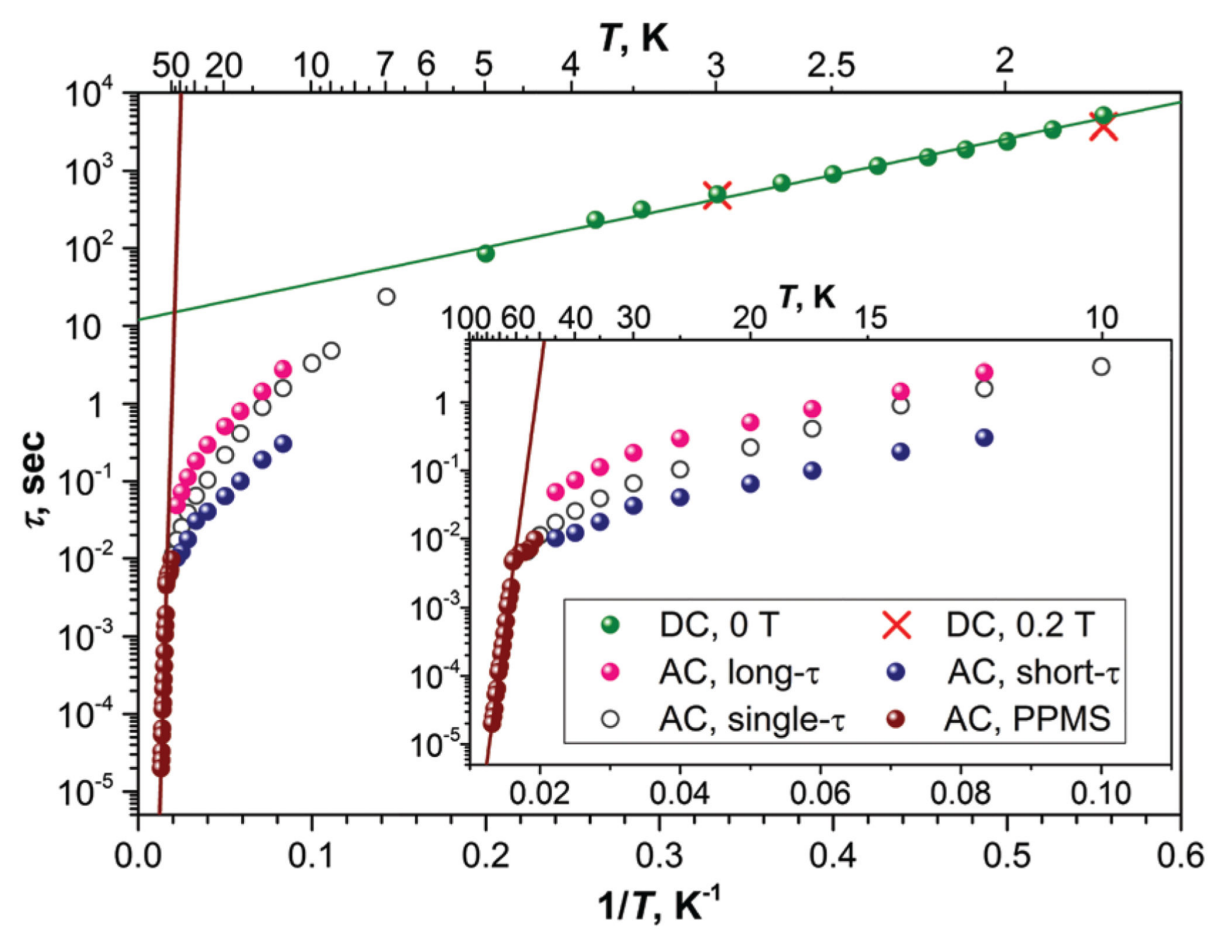
Relaxation times of the magnetization of Dy_2_ScN@C_80_. Green dots denote the values from dc measurements in zero field; two in-field points (red crosses) are also shown. AC values are measured with MPMS XL (7–50 K; open, magenta, and blue dots) and with PPMS (brown dots, 52–76 K). Magenta and blue dots denote long and short times from double-τ fits of the ac data, respectively, open dots – for single-τ fits.

**Fig. 4:**
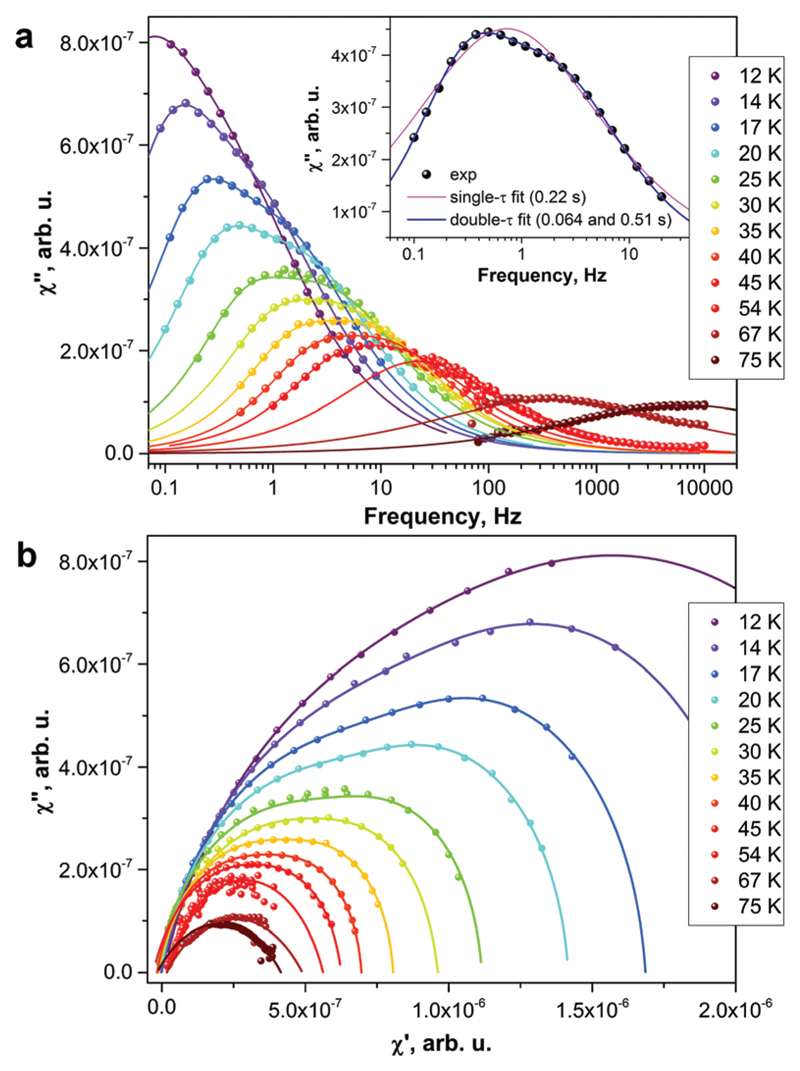
AC-susceptibility measurements of Dy_2_ScN@C_80_ at selected temperatures: (a) *χ*″ and (b) Cole–Cole plots. Dots are experimental points, lines are fits to the points with generalized Debye model with either one or two relaxation times. The inset in (a) shows the fitting of the 20 K data with double-τ and single-τ models.

**Fig. 5:**
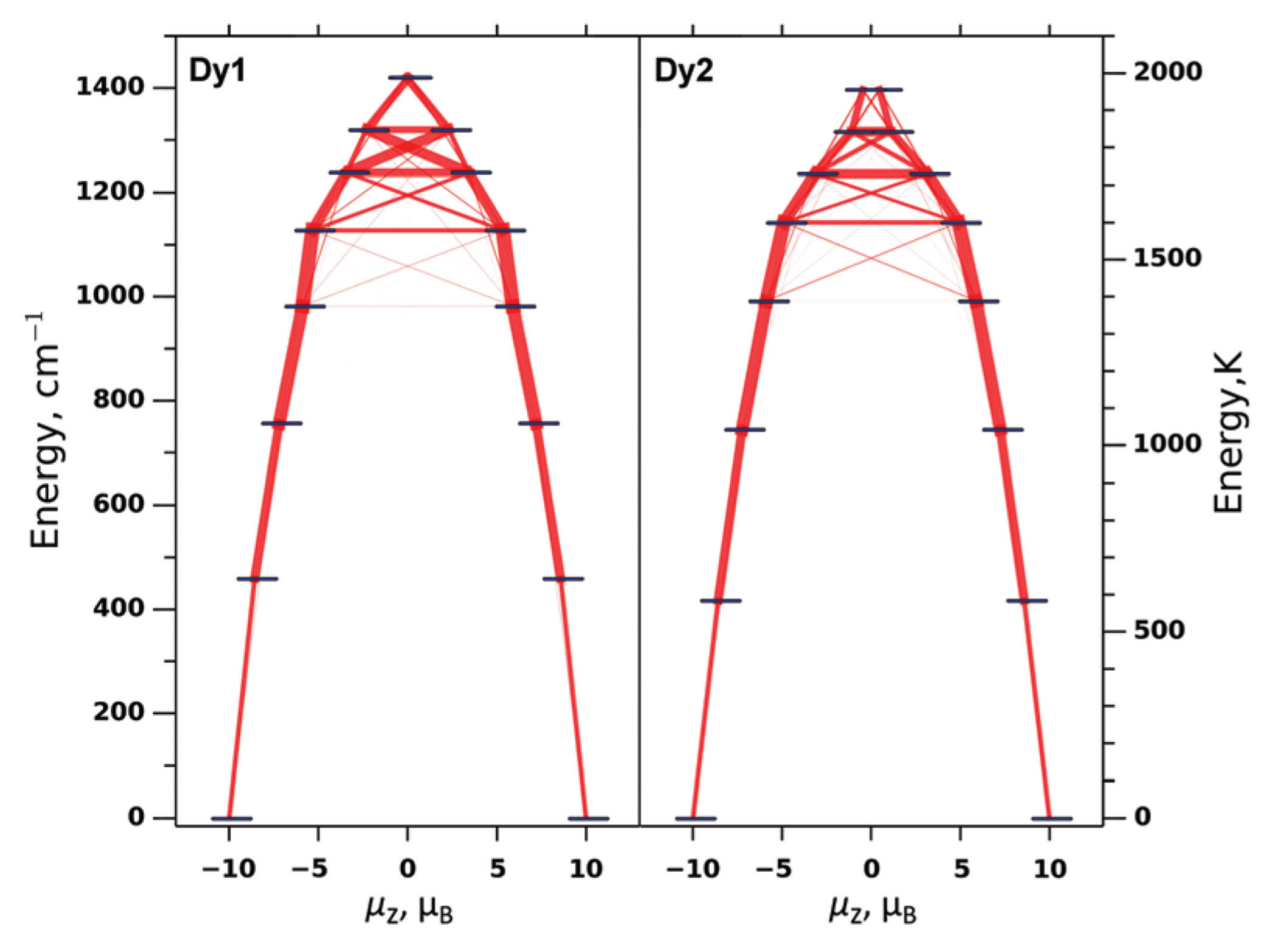
The energies of single-ion CF states of Dy1 and Dy2 as computed *ab initio* at the CASSCF/RASSI level. Red lines visualize transition probabilities computed from transverse components of the *g*-tensor (the thicker the line – the higher the probability).

## Footnotes

§ After the two solutions diffused together over a period of one month, small black crystals (0.2 × 0.1 × 0.1 mm^3^) formed. X-ray diffraction data collection was carried out at 100 K at the BESSY storage ring (BL14.3, Berlin-Adlershof, Germany)^23^ using a MAR225 CCD detector, *λ* = 0.89429 Å. Diffraction data was processed with XDSAPP2.0 suite.^24^ The structure was solved by direct methods and refined using all data (based on F^2^) by SHELX 2016.^25^ Hydrogen atoms were located in a difference map, added geometrically, and refined with a riding model. The data can be obtained free of charge from The Cambridge Crystallographic Data Centre with CCDC no. 1547067.

¶ PBE/TZ2P level, Priroda code.^26^
