# Supplementary material for: Record-high thermal barrier of the relaxation of magnetization in the nitride clusterfullerene Dy2ScN@C80-Ih†
Source: Chem Commun (Camb). Author manuscript; Available in PMC 2017 Dec 14. (PMC5730045; doi:10.1039/c7cc03580b)
Supplement: ESI [file NIHMS73593-supplement-ESI.pdf]

Electronic Supplementary Material (ESI) for ChemComm.
This journal is © The Royal Society of Chemistry 2017

Record-high thermal barrier of the relaxation of magnetization in the nitride clusterfullerene $Dy_2ScN@C_{80}$-I_h

D. S. Krylov,[$] F. Liu,[$] S. M. Avdoshenko, L. Spree, B. Weise, A. Waske, A. U. B. Wolter, B. Büchner and A. A. Popov*

Supporting Information

S1. Synthesis and Separation of $Dy_2ScN@C_{80}$ S2

S2. X-ray Crystallographic Analysis of $Dy_2ScN@C_{80}$ S6

S3. Configuration of the M_2ScN cluster in $M_2ScN@C_{80}$-I_h: experiment versus theory S9

S4. DC magnetometry and relaxation times S11

S5. Ab initio calculations S12

S6. Coupling between magnetic moments of Dy ions in $Dy_2ScN@C_{80}$ S16

S7. References S20

S8. DFT-optimized Cartesian coordinates of $Y_2ScN@C_{80}$ conformers S21

S1. Synthesis and Separation of $Dy_2ScN@C_{80}$

$Dy_2ScN@C_{80}$ was synthesized in a modified Krätschmer-Huffman fullerene generator by vaporizing composite graphite rods (ϕ 6 × 100 mm) containing a mixture of Dy_2O_3, Sc_2O_3, guanidine thiocyanate (GT, as solid nitrogen source[1]) and graphite powder with the addition of 180 mbar He as described previously.[2] To investigate the effect of Dy-Sc ratio on the formation of $Dy_{3-x}Sc_xN@C_{80}$ (x=0-3), syntheses by using mixture of Dy_2O_3, Sc_2O_3, GT and graphite powder with different molar ratios of Dy-Sc were carried out. The as-produced soot was Soxhlet-extracted by CS_2 for 24 h, and the resulting brown-yellow solution was distilled to remove CS_2 and then immediately re-dissolved in toluene and subsequently passed through a 0.2 μm Telflon filter for HPLC separation. The isolation of $Dy_2ScN@C_{80}$ was performed by multi-step HPLC. The purities of the isolated $Dy_2ScN@C_{80}$ was further checked by laser desorption/ionization time-of-flight (LDI-TOF) mass spectroscopic (MS) analysis (Bruker autoflex).

Figure S1 shows the HPLC profiles of the fullerene mixture extracted from Dy_2O_3-Sc_2O_3-GT with different Dy-Sc ratios. The MS spectra were shown in Figure S2. The result indicates that the relative yield of $Dy_2ScN@C_{80}$ is enhanced with the reducing of Dy-Sc ratio, a ratio of Dy:Sc:GT:C=1:0.5:2.5:7.5 is recommended for the higher selectivity in the synthesis of $Dy_2ScN@C_{80}$.

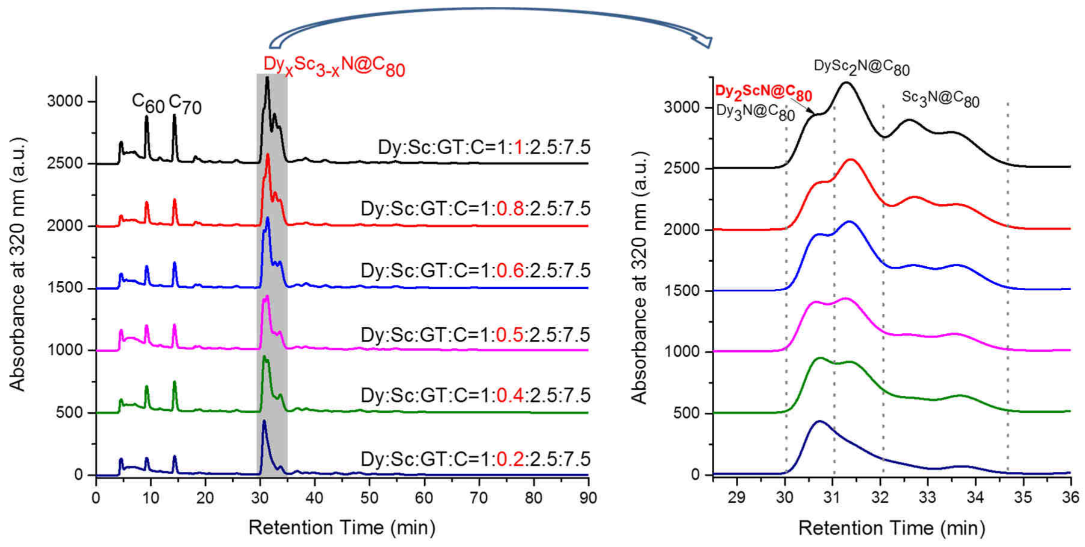

Figure S1. HPLC profiles of the fullerene mixture extracted from Dy_2O_3-Sc_2O_3-GT with different Dy-Sc ratios. HPLC conditions: linear combination of two 4.6 × 250 mm Buckyprep columns; flow rate 1.6 mL/min; injection volume 800 μL; toluene as eluent; 40 °C. As shown in the enlarged figure, the left peak mainly contains $Dy_2ScN@C_{80}$ (I), $Dy_3N@C_{80}$ (I), $Dy_2ScN@C_{78}$, and $Sc_3N@C_{78}$; The middle peak mainly contains $DySc_2N@C_{80}$ (I); The right peak mainly contains $Sc_3N@C_{80}$ (I, II), and $Dy_xSc_{3-x}N@C_{80}$ (II, x=1,2).

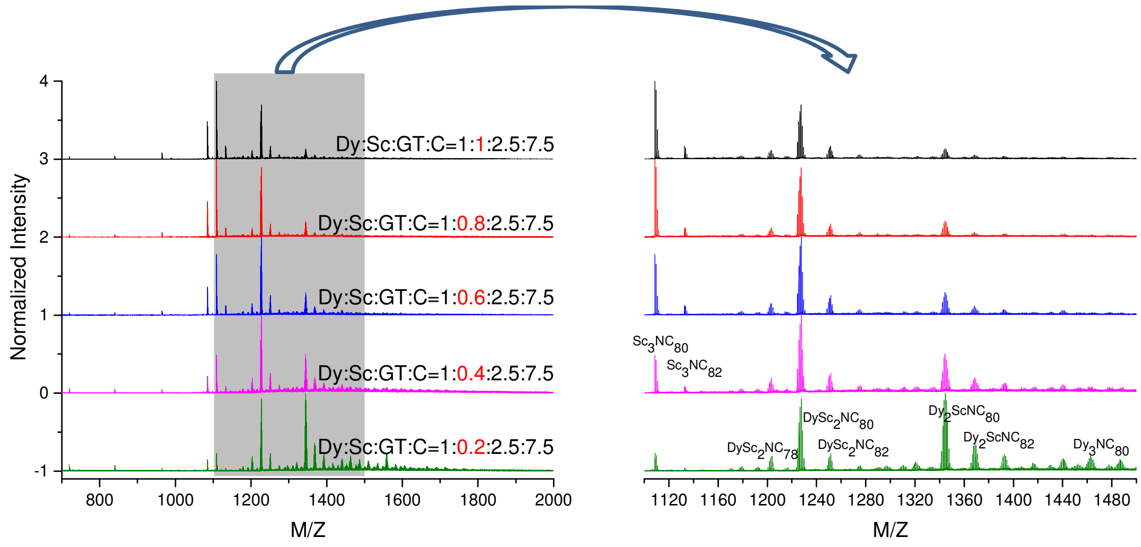

Figure S2. Positive-ion laser desorption/ionization time-of flight (LDI-TOF) mass spectra of the fullerene mixture extracted from Dy_2O_3-Sc_2O_3-GT with different Dy-Sc ratios.

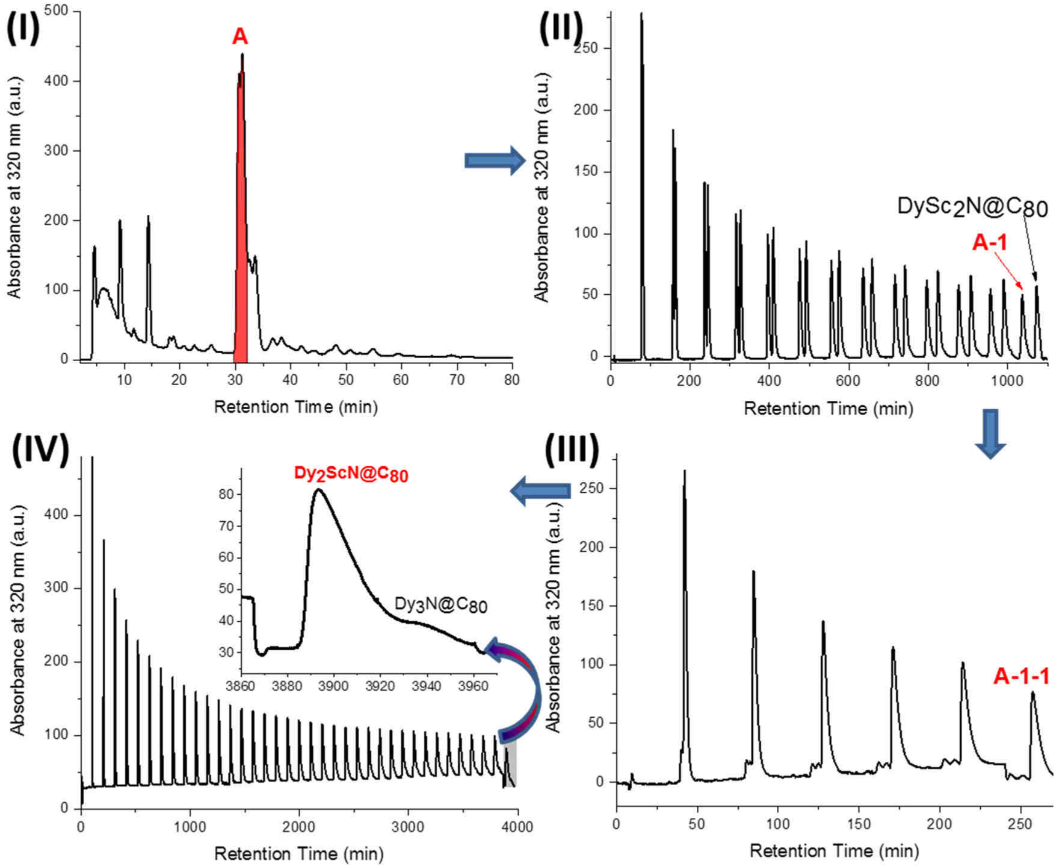

Figure S3. Separation of $Dy_2ScN@C_{80}$. Four steps were required to obtain pure $Dy_2ScN@C_{80}$ from the extracted fullerene mixture. **(I)** HPLC profile of the fullerene mixture extracted from Dy_2O_3-Sc_2O_3- GT. Fraction A contains the target $Dy_2ScN@C_{80}$. (HPLC conditions: linear combination of two 4.6 × 250 mm Buckyprep columns; flow rate 1.6 mL/min; injection volume 800 μL; toluene as eluent; 40 °C) **(II)** Recycling HPLC profile of fraction A (10 × 250 mm Buckyprep column; flow rate 2 mL/min; injection volume 4.5 mL; toluene as eluent). **(III)** Recycling HPLC profile of fraction A-1 (10 × 250 mm Buckyprep-M column; flow rate 2 mL/min; injection volume 4.5 mL; toluene as eluent). **(IV)** Recycling HPLC profile of fraction A-1-1 (10 × 250 mm Buckyprep column; flow rate 1 mL/min; injection volume 4.5 mL; toluene as eluent). Pure $Dy_2ScN@C_{80}$ was obtained.

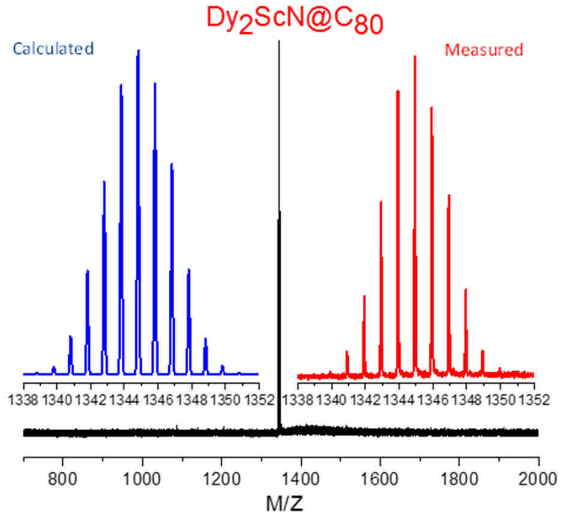

Figure S4. Positive-ion laser desorption/ionization time-of flight (LDI-TOF) mass spectrum of the purified $Dy_2ScN@C_{80}$. Insets: measured and calculated isotopic distributions of $Dy_2ScN@C_{80}$.

S2. X-ray Crystallographic Analysis of $Dy_2ScN@C_{80}$

Crystal growth of $Dy_2ScN@I_h(7)$-$C_{80}\cdot Ni^{II}(OEP)\cdot 2(C_6H_6)$ was accomplished by layering benzene solution of Ni^{II}(OEP) over a solution of $Dy_2ScN@I_h(7)$-C_{80} in CS_2. After the two solutions diffused together over a period of one month, small black crystals (0.2 x 0.1 x 0.1 mm^3) suitable for X-ray crystallographic study formed. X-ray diffraction data collection for the crystal was carried out at 100 K at the BESSY storage ring (BL14.3, Berlin-Adlershof, Germany)[3] using a MAR225 CCD detector, λ = 0.89429 Å. Processing diffraction data was done with XDSAPP2.0 suite.[4] The structure was solved by direct methods and refined using all data (based on F^2) by SHELX 2016.[5] Hydrogen atoms were located in a difference map, added geometrically, and refined with a riding model. The data can be obtained free of charge from The Cambridge Crystallographic Data Centre with CCDC No. 1547067.

Figure S5 shows the location of the main site of the Dy_2ScN cluster in the $I_h(7)$-C_{80}. Figure S6 depicts the geometric structure of the main site of Dy_2ScN and the interactions of the Dy/Sc atoms with the closest portions of the cage. The bond lengths are: 2.078(6) Å, 1.965(6) Å, and 1.960(7) Å for Dy1-N1, Dy2-N1, and Sc1-N1, respectively. Notice that because of the vicinity of Dy2 and Sc2, Dy4 and Sc1, and the huge different diffraction powers of Dy and Sc atoms, it is hard to separate the model for Dy and Sc at these sites, so the bond length of Dy2-N1 and Sc1-N1 is not reliable. The bond angles of Dy1-N1-Sc1, Dy1-N1-Dy2, and Dy2-N1-Sc1 are 121.1(3)°, 125.7(3)°, and 112.1(3)°, respectively. The sum of these bond angles is 359°, indicates that the encapsulated Dy_2ScN cluster is planar.

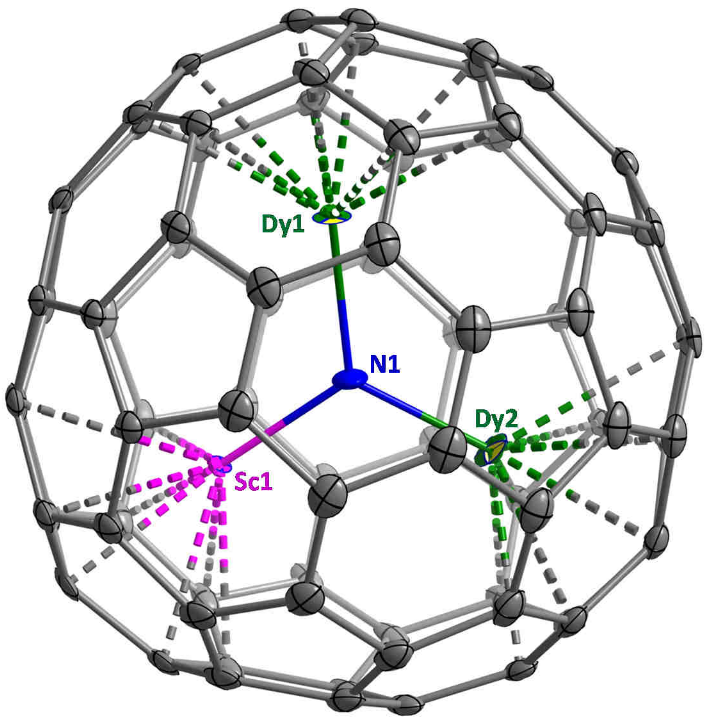

Figure S5. Drawing showing the $I_h(7)$-C_{80} fullerene cage with the major Dy_2ScN site. Displacement parameters are shown at the 30% probability level.

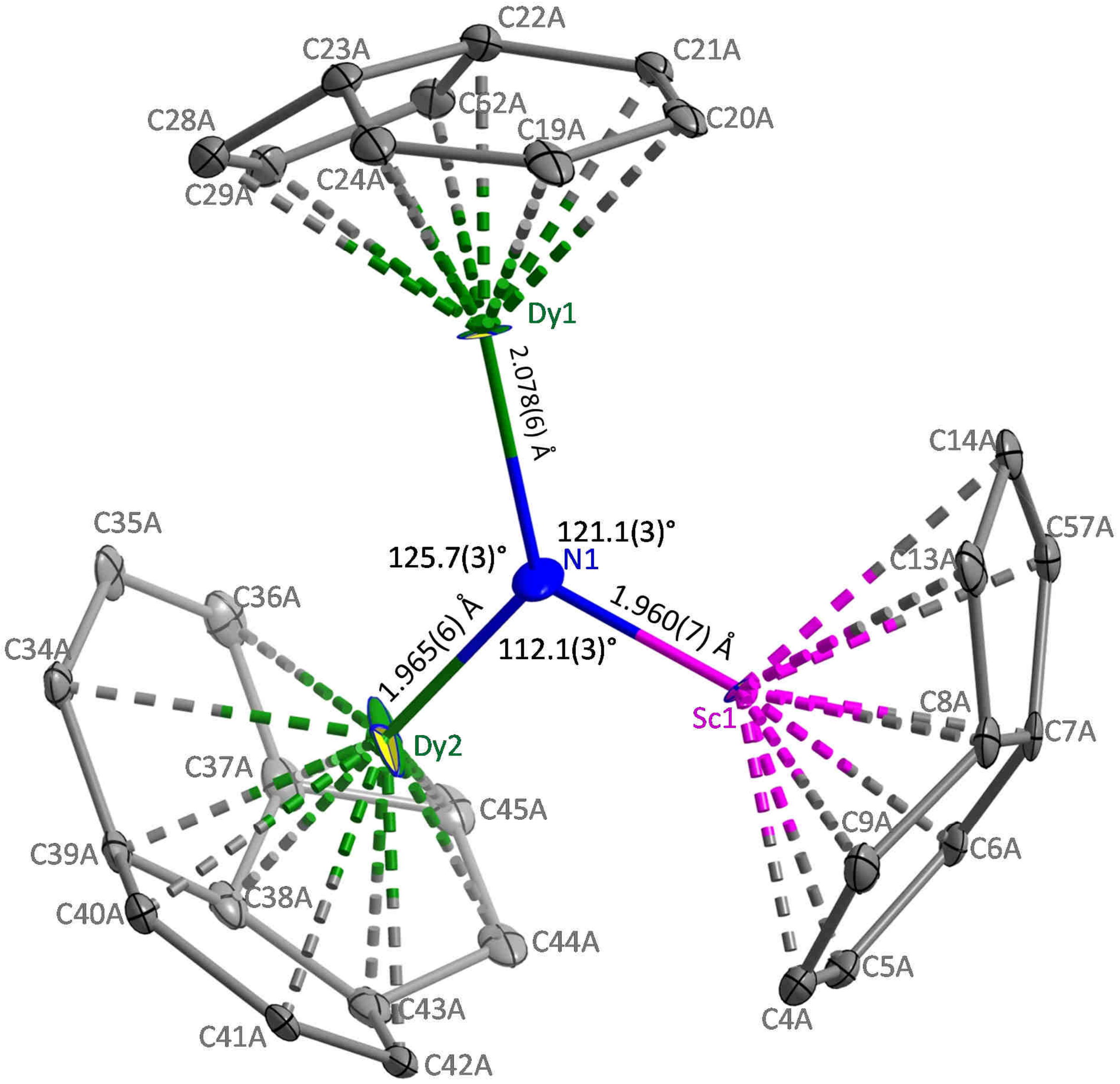

Figure S6. Major site of Dy_2ScN cluster within the I_h(7)-C_{80} cage with X-ray determined bond lengths, bond angles and the interactions of the Dy atoms with the closest portions of the cage are shown. Displacement parameters are shown at the 30% probability level. Due to the disorder in the crystal (in particular, the presence of another Dy_2ScN cluster orientation with close almost overlapping Dy and Sc positions), bond lengths in the structure seem to be not very reliable as evidenced, for instance, by the very short Dy2-N1 bond (it can be compared to the Dy1-N1 bond length, which has expected bond length).

Table S1. Crystal data and data collection parameters.

Crystal	$Dy_2ScN@I_h(7)\text{-}C_{80}\cdot Ni^{II}(OEP)\cdot 2(C_6H_6)$
Formula	C128 H56 Dy2 N5 Ni Sc
Formula weight	2092.44
Color, habit	Black, block
Crystal system	triclinic
Space group	*P-1*
a, Å	14.700(3)
b, Å	15.380(3)
c, Å	17.710(4)
α, deg	81.08(3)
β, deg	74.46(3)
γ, deg	86.41(3)
Volume, $Å^3$	3810.0(15)
Z	2
T, K	100
Radiation (λ, Å)	Synchrotron Radiation (0.89429)
Unique data (R_{int})	14242 (0.0744)
Parameters	1238
Restraints	1152
Observed data ($I > 2\sigma(I)$)	12882
$R1^a$ (observed data)	0.0923
$wR2^b$ (all data)	0.2458

^{a}For data with $I > 2\sigma(I)$, $R1=1||F_O|-|F_C||/\sum|F_O|$. ^{b}For all data, $wR2 =\{\sum[w(F_O{}^2-F_C{}^2)^2]\}/\sum[w(F_O{}^2)^2]\}^{1/2}$.

S3. Configuration of the M_2ScN cluster in $M_2ScN@C_{80}$-I_h: experiment versus theory

Extended DFT studies of $M_3N@C_{80}$-I_h molecules with different metals (M = Sc, Y, Lu) reported earlier showed that the M_3N cluster has two especially stable configurations (conformers) inside the C_{80}-I_h fullerene cage with C_3 and C_s symmetry.[6, 7] The structures of the conformers are shown in Figs. S7 and S8 for $Y_3N@C_{80}$-I_h (as DFT computations with Dy are severely complicated and because Y has similar ionic radius and chemical properties to Dy, we use Y as model for Dy). In the C_3-conformer, all metal atoms are equivalent and have quasi-η^6 coordination to one of the cage the hexagons (metal atoms is somewhat displaced from the center of the hexagon towards one of the pentagon/hexagon edges). In the C_s conformer, two metal atoms have similar quasi-η^6 coordination as in the C_3-conformer, whereas the third metal atom on the symmetry plane is coordinated to the carbon atom on the pentagon/hexagon/hexagon junction. At the PBE/TZ2P level of theory, C_3 conformer is more stable than Cs-conformer of $M_3N@C_{80}$-I_h by 4.6 and 8.8 kJ/mol for M = Sc and Y, respectively.

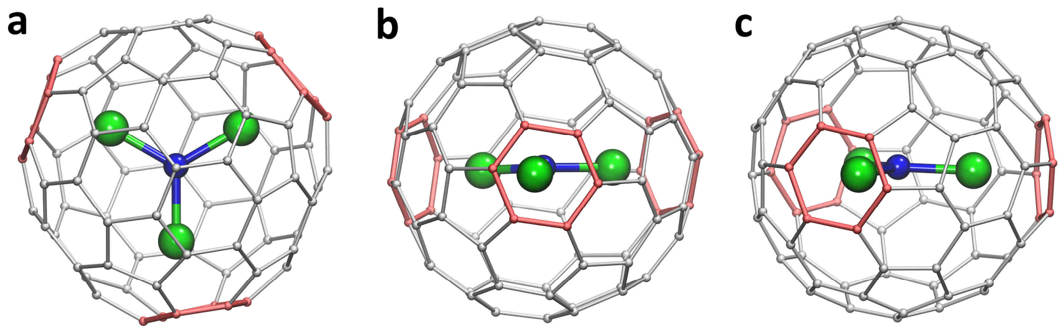

Figure S7. DFT-optimized molecular structure of $Y_3N@C_{80}$ with C_3-symmetric configuration. The molecule is shown in three projections, C_3 axis is perpendicular to the plane of the cluster and passes through nitrogen and two carbon atoms of the cage. Hexagons with quasi-η^6 coordination of Y atoms are shown in pink, other cage carbons are grey. Y–N bond length is 2.060 Å, the Y_3N cluster is slightly pyramidal (nitrogen is 0.083 Å above the plane of metal atoms).

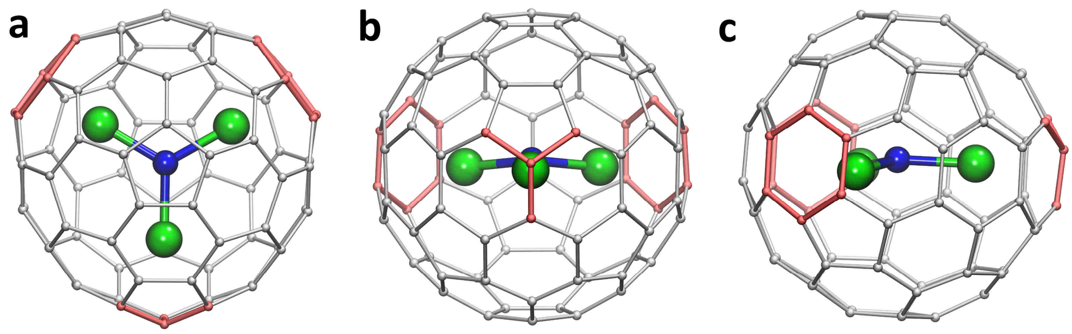

Figure S8. DFT-optimized molecular structure of $Y_3N@C_{80}$ with C_s-symmetric configuration. The molecule is shown in three projections, symmetry plane is perpendicular to the plane of the cluster and passes through nitrogen and one of the Y atoms. Hexagons with quasi-η^6 coordination of Y as well as the fragment of the cage with close Y-C contacts are shown in pink, other cage carbons are grey. Y-N bond lengths are 2.064 Å and 2×2.066 Å, the Y_3N cluster is more pyramidal than in the C_3-symmetric conformer (nitrogen is 0.199 Å above the plane of metal atoms).

Substitution of one of the Y atoms in C_3-$Y_3N@C_{80}$ by Sc leads to one possible conformer of $Y_2ScN@C_{80}$. C_s-$Y_3N@C_{80}$ has two non-equivalent Y atoms and hence can lead to two conformers of $Y_2ScN@C_{80}$. However, in the course of DFT optimization, both C_s-derived conformers of $Y_2ScN@C_{80}$ converged to the same structure. Thus, we obtained two conformers of $Y_2ScN@C_{80}$-I_h, one derived from the C_3-symmetric arrangement of the M_3N cluster inside the cage, and another one derived from the C_s-configuration. At the PBE/TZ2P level, the former is more stable by 6.3 kJ/mol. Both structures are shown in Figure S9 in comparison to the Dy_2ScN cluster sites determined experimentally in $Dy_2ScN@C_{80}$-I_h. As can be seen, experimental configurations of the cluster correspond reasonably well to DFT-optimized ones. Furthermore, the site with higher occupancy in the X-ray structure corresponds to the most stable conformer of $Y_2ScN@C_{80}$. It shows that disorder in the experimental structure has intrinsic thermodynamic reasons caused by co-existence of similar low-energy conformations of the cluster inside the fullerene cage.

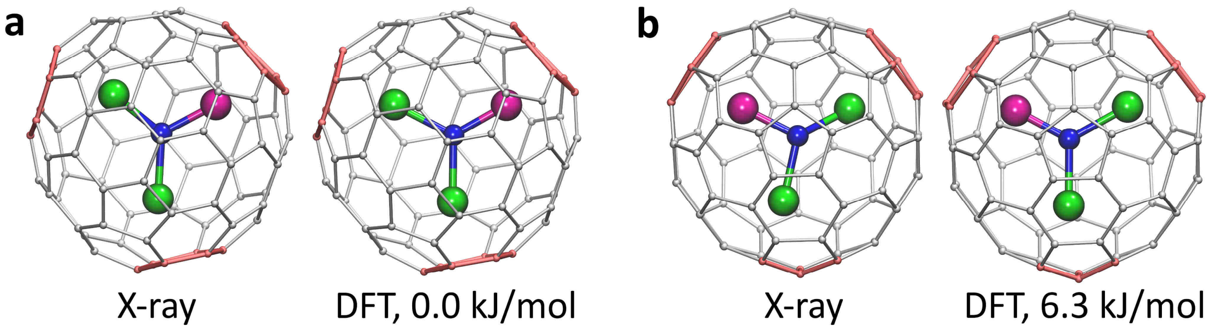

Figure S9. Comparison of experimental (X-ray) structures of $Dy_2ScN@C_{80}$ and corresponding DFT-optimized molecular structures of $Y_2ScN@C_{80}$: **(a)** Main configuration of the Dy_2ScN cluster (occupancy 0.69) corresponds to the conformer of $Y_2ScN@C_{80}$ derived from the C_3-conformer of $Y_3N@C_{80}$; **(b)** Minor configuration of the Dy_2ScN cluster (occupancy 0.31) corresponds to the conformer of $Y_2ScN@C_{80}$ derived from the C_s-conformer of $Y_3N@C_{80}$. Note that the energy difference between C_3- and C_s-derived conformers of $Y_2ScN@C_{80}$ is 6.3 kJ/mol in favor of the C_3-derived structure, which correlates with the higher occupancy of the corresponding configuration in $Dy_2ScN@C_{80}$.

S4. DC magnetometry and relaxation times

DC magnetization measurements were performed using a Quantum Design VSM MPMS3 magnetometer. The sample drop-casted from CS_2 solution into a standard powder sample holder (note that co-crystallization with Ni^{II}(OEP) was used only for X-ray diffraction studies, whereas magnetic properties were studied for the pristine $Dy_2ScN@C_{80}$). To measure relaxation time in dc mode, the sample was first magnetized to the saturation at 5 T, then the field was swept as fast to B = 0 T or B = 0.2 T, and then the decay of magnetization was recorded. Decay curves were fitted using stretched exponential function:

$$Y_{stretched} = A_0 e^{-\left(\frac{t}{\tau}\right)^{\beta}} + y_0$$

where τ is the relaxation time and y_0 is an equilibrium magnetization at the given field and temperature.

Table S2. Magnetization relaxation times determined from the stretched exponential fitting of zero-field DC relaxation measurements.

T, K	τ, sec	St. dev., sec	β
1.8	5107.44	72.506	0.65
1.9	3358.05	16.431	0.66
2	2357.28	10.202	0.69
2.1	1871.55	3.910	0.69
2.2	1491.76	2.841	0.71
2.35	1147.97	0.897	0.72
2.5	899.69	0.591	0.74
2.7	696.24	0.260	0.74
3	496.47	0.207	0.76
3.45	316.06	0.076	0.77
3.8	232.65	0.251	0.80
5	84.92	0.113	0.86
7	23.67	0.045	0.95

S5. Ab initio calculations

Ab initio energies and wave functions of CF multiplets for the $Dy_2ScN@C_{80}$ molecule have been calculated using the quantum chemistry package MOLCAS 8.0. Each Dy(III) atom in the system was treated independently, while the second Dy ion was substituted by f-electron free Yttrium. Single point complete active space self-consistent field with spin-orbit interactions calculations (CASSCF/SO-RASSI) level of theory was employed to derive ab initio values (Tables S3-S5). The maximum ground state J = 15/2 results in eight low-lying Kramers doublets. The active space of the CASSCF calculations includes eleven active electrons and the seven active orbitals (e.g. CAS (11,7)). All 21 sextet states and 224 quartets and only 490 doublets were included in the state-averaged CASSCF procedure and were further mixed by spin-orbit coupling in the RASSI procedure. VDZ quality atomic natural extended relativistic basis set (ANO-RCC) was employed. The single ion magnetic properties and CF parameters were calculated with use of SINGLE_ANISO module. The CFs were used to construct a model Zeeman Hamiltonian with |J,m_J> basis. Based on this Hamiltonian transition probabilities were estimated using PHI code. The ground magnetic state of both Dy ions is J_z = ±15/2, orientation the anisotropy axes in the Dy_2ScN cluster is depicted in Figure S10.

Table S3: CASSCF/SO-RASSI/SINGLE_ANISO calculations results summary for the $Dy_2ScN@C_{80}$ molecule. G-tensors components and energies of the eight low lying KDs of the Dy1. The Θ-angle shows the rotation of the state vectors with respect to the ground state.

state	Leading m_J term (%)	g_x	g_y	g_z	E, cm^{-1}	Θ, °
1	99.9 |15/2⟩	0.0000	0.0001	19.8785	0.0	0.0
2	93.9 |13/2⟩	0.0039	0.0043	17.1703	457.1	10.6
3	81.1 |11/2⟩	0.0691	0.0789	14.3218	754.8	10.9
4	74.2 |9/2⟩	1.1260	1.3284	11.3961	980.0	1.5
5	69.8 |7/2⟩	3.2091	4.0556	9.5690	1124.4	34.4
6	63.0 |5/2⟩	0.1966	3.8837	9.5904	1234.3	69.3
7	56.0 |3/2⟩	2.1280	4.3568	12.2452	1319.6	88.2
8	42.0 |1/2⟩	0.3556	1.1934	18.1672	1422.7	94.6

Table S4: CASSCF/SO-RASSI/SINGLE_ANISO calculations results summary for the $Dy_2ScN@C_{80}$ molecule. G-tensors components and energies of the eight low lying KDs of the Dy2. The Θ-angle shows the rotation of the state vectors with respect to the ground state.

state	Leading m_J term (%)	g_x	g_y	g_z	E, cm^{-1}	Θ, °
1	99.7 |15/2⟩	0.0001	0.0001	19.8679	0.0	0.0
2	95.0 |13/2⟩	0.0017	0.0020	17.1476	417.5	9.7
3	85.9 |11/2⟩	0.0846	0.0984	14.2677	741.3	8.0
4	81.0 |9/2⟩	0.6415	0.7382	11.4081	989.0	3.3
5	76.0 |7/2⟩	3.2286	3.3287	9.1099	1140.7	30.4
6	64.1 |5/2⟩	2.2116	3.9233	10.1780	1233.0	106.0
7	31.9 |3/2⟩	1.5964	2.7165	13.3475	1316.3	92.6
8	48.4 |1/2⟩	0.3963	1.4520	17.9062	1399.3	82.5

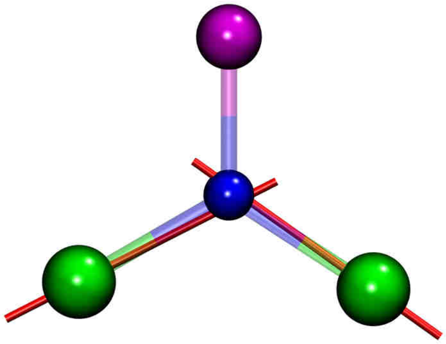

Figure S10. Magnetic anisotropy axes (red lines) for each Dy center in $Dy_2ScN@C_{80}$ according to *ab initio* calculations. Note the slight deviation of the axes from Dy-N bonds. Color code: Dy – green, Sc – magenta, N – blue

Table S5.
Ab-intio derived (SINGLE_ANISO) crystal field parameters in Stevens Notation B(q,k)(cm^{-1}) of Dy_2ScNC_{80} *molecules*.

**index**		Dy1	Dy2
**k**	**q**	**B(q,k)**	**B(q,k)**
2	-2	1.40E+00	-7.97E-01
2	-1	2.33E+00	2.20E+00
2	0	-7.80E+00	-7.86E+00
2	1	1.16E+00	-1.02E-01
2	2	-1.72E-01	-1.05E+00
4	-4	6.36E-04	2.43E-03
4	-3	-4.73E-03	-2.48E-03
4	-2	-6.59E-04	-8.53E-04
4	-1	4.37E-03	6.41E-03
4	0	-4.51E-03	-4.79E-03
4	1	1.64E-03	-1.27E-03
4	2	1.73E-03	2.67E-03
4	3	-1.79E-03	-9.42E-03
4	4	-1.26E-03	2.68E-04
6	-6	-1.60E-04	-3.90E-05
6	-5	6.66E-04	-8.58E-05
6	-4	2.43E-05	1.96E-04
6	-3	-2.24E-04	-3.06E-05
6	-2	-2.15E-05	-1.85E-05
6	-1	-3.92E-04	-4.42E-04
6	0	-2.11E-05	1.70E-07
6	1	-1.85E-04	2.73E-05
6	2	-1.30E-05	8.82E-05
6	3	3.10E-05	-3.22E-04
6	4	-1.37E-04	-7.85E-06
6	5	2.25E-05	1.01E-03
6	6	1.05E-04	3.10E-05

Table S6. Transition probabilities between different single-ion KD states computed for Dy1 (the values less than 0.01 are not shown)

	$\lvert 1^+\rangle$	$\lvert 1^-\rangle$	$\lvert 2^+\rangle$	$\lvert 2^-\rangle$	$\lvert 3^+\rangle$	$\lvert 3^-\rangle$	$\lvert 4^+\rangle$	$\lvert 4^-\rangle$	$\lvert 5^+\rangle$	$\lvert 5^-\rangle$	$\lvert 6^+\rangle$	$\lvert 6^-\rangle$	$\lvert 7^+\rangle$	$\lvert 7^-\rangle$	$\lvert 8^+\rangle$	$\lvert 8^-\rangle$
$\langle^+1\rvert$			4.16		0.28		0.02									
$\langle^-1\rvert$				4.16		0.28		0.02								
$\langle^+2\rvert$	4.16				7.97		0.04		0.03		0.01				0.01	
$\langle^-2\rvert$		4.16				7.97		0.04		0.03		0.01				0.01
$\langle^+3\rvert$	0.28		7.97				11.39		0.13	0.04	0.09	0.01			0.04	
$\langle^-3\rvert$		0.28		7.97				11.39	0.04	0.13	0.01	0.09				0.04
$\langle^+4\rvert$	0.02		0.04		11.39			0.26	12.84	0.38	1.02	0.04	0.22	0.04	0.05	
$\langle^-4\rvert$		0.02		0.04		11.39	0.26		0.38	12.84	0.04	1.02	0.04	0.22		0.05
$\langle^+5\rvert$			0.03		0.13	0.04	12.84	0.38		4.08	9.61	3.37	0.91	0.34	0.21	0.04
$\langle^-5\rvert$				0.03	0.04	0.13	0.38	12.84	4.08		3.37	9.61	0.34	0.91	0.04	0.21
$\langle^+6\rvert$			0.01		0.09	0.01	1.02	0.04	9.61	3.37		7.14	9.45	4.35	1.03	0.13
$\langle^-6\rvert$				0.01	0.01	0.09	0.04	1.02	3.37	9.61	7.14		4.35	9.45	0.13	1.03
$\langle^+7\rvert$							0.22	0.04	0.91	0.34	9.45	4.35		6.11	7.01	0.76
$\langle^-7\rvert$							0.04	0.22	0.34	0.91	4.35	9.45	6.11		0.76	7.01
$\langle^+8\rvert$			0.01		0.04		0.05		0.21	0.04	1.03	0.13	7.01	0.76		1.40
$\langle^-8\rvert$				0.01		0.04		0.05	0.04	0.21	0.13	1.03	0.76	7.01	1.40	

Table S7. Transition probabilities between different single-ion KD states computed for Dy2 (the values less than 0.01 are not shown)

	$\vert 1^+\rangle$	$\vert 1^-\rangle$	$\vert 2^+\rangle$	$\vert 2^-\rangle$	$\vert 3^+\rangle$	$\vert 3^-\rangle$	$\vert 4^+\rangle$	$\vert 4^-\rangle$	$\vert 5^+\rangle$	$\vert 5^-\rangle$	$\vert 6^+\rangle$	$\vert 6^-\rangle$	$\vert 7^+\rangle$	$\vert 7^-\rangle$	$\vert 8^+\rangle$	$\vert 8^-\rangle$
$\langle ^+1\vert$			4.23		0.23		0.03									
$\langle ^-1\vert$				4.23		0.23		0.03								
$\langle ^+2\vert$	4.23				8.04		0.01		0.04		0.02		0.01		0.02	
$\langle ^-2\vert$		4.23				8.04		0.01		0.04		0.02		0.01		0.02
$\langle ^+3\vert$	0.23		8.04				11.38	0.01	0.11	0.02	0.14		0.01		0.05	
$\langle ^-3\vert$		0.23		8.04			0.01	11.38	0.02	0.11		0.14		0.01		0.05
$\langle ^+4\vert$	0.03		0.01		11.38	0.01		0.10	12.41	0.83	0.90	0.13	0.29	0.08	0.06	0.01
$\langle ^-4\vert$		0.03		0.01	0.01	11.38	0.10		0.83	12.41	0.13	0.90	0.08	0.29	0.01	0.06
$\langle ^+5\vert$			0.04		0.11	0.02	12.41	0.83		4.16	10.72	2.94	1.43	0.17	0.31	0.05
$\langle ^-5\vert$				0.04	0.02	0.11	0.83	12.41	4.16		2.94	10.72	0.17	1.43	0.05	0.31
$\langle ^+6\vert$			0.02		0.14		0.90	0.13	10.72	2.94		8.01	7.58	4.18	1.21	0.05
$\langle ^-6\vert$				0.02		0.14	0.13	0.90	2.94	10.72	8.01		4.18	7.58	0.05	1.21
$\langle ^+7\vert$			0.01		0.01		0.29	0.08	1.43	0.17	7.58	4.18		8.67	6.39	1.77
$\langle ^-7\vert$				0.01		0.01	0.08	0.29	0.17	1.43	4.18	7.58	8.67		1.77	6.39
$\langle ^+8\vert$			0.02		0.05		0.06	0.01	0.31	0.05	1.21	0.05	6.39	1.77		0.49
$\langle ^-8\vert$				0.02		0.05	0.01	0.06	0.05	0.31	0.05	1.21	1.77	6.39	0.49	

S6. Coupling between magnetic moments of Dy ions in $Dy_2ScN@C_{80}$

The system with two Dy centers with magnetic moments J_1 and J_2 weakly coupled through exchange/dipolar interaction can be described by the following effective spin Hamiltonian:

$$\hat{H}_{tot} = \hat{H}_{CF1} + \hat{H}_{CF2} - 2j_{12}\hat{J}_1 \cdot \hat{J}_2 \qquad \text{(S1)}$$

where the H_{CFi} terms are single-ion crystal-field Hamiltonians, and the last term describes the exchange and dipolar interactions between two Dy centers (rather unfortunately, both exchange coupling and the total magnetic moment of lanthanide are traditionally designated as *J*, so we use the small letter *j* for the coupling and the capital *J* for the momentum). In the spirit of the Lines model, both exchange and dipolar interactions are modelled here by a single isotropic coupling parameter j_{12}. In the simulation discussed below, the CF parameters in Eq. S1 and the angle between the main axes of the magnetization of individual Dy centres are obtained from *ab initio* calculations as described in the previous section, and simulations are performed using the PHI code. To match the experimental energy of the first exchange/dipolar excited state (estimated as 10.7 K from the Arrhenius behavior of the low-temperature relaxation times), the absolute value of the exchange/dipolar parameter j_{12} should be equal to 0.073 cm^{-1}. The sign of j_{12} (and hence the nature of the coupling between the magnetic moments of Dy centers) can be determined from the temperature dependence of χT (Fig. S11) as well as from the magnetization curves (Fig. S12).

By definition, the magnetic susceptibility χ is the derivative of the magnetization M with respect to the magnetic field B, whereas the experimentally measured quantity is the ratio M/B. For small values of B, the ratio and the derivative are quite close, but with the increase of the field a deviation between both quantities can become significant. Therefore, we will use the designation (M/B)T for all experimental curves.

When measured in small fields (0.2 T, 0.5 T, 1 T), the experimental *(M/B)T* curves show a sharp peak at low temperature, which becomes smaller as the external field is increasing (Fig. S11a). At B = 3 T, the peak is not observed anymore. The same pattern is observed in simulated χT curves for the ferromagnetically-coupled system (j_{12} = +0.073 cm^{-1}). When the magnetic moments of Dy ions are coupled antiferromagnetically (j_{12} = −0.073 cm^{-1}), *χT* exhibits a gradual increase without a low-temperature peak at any value of the external field (Fig. S11c). Thus, Figure S11 shows that the experimental *(M/B)T* pattern is reproduced well by the simulation for the ferromagnetically coupled system.

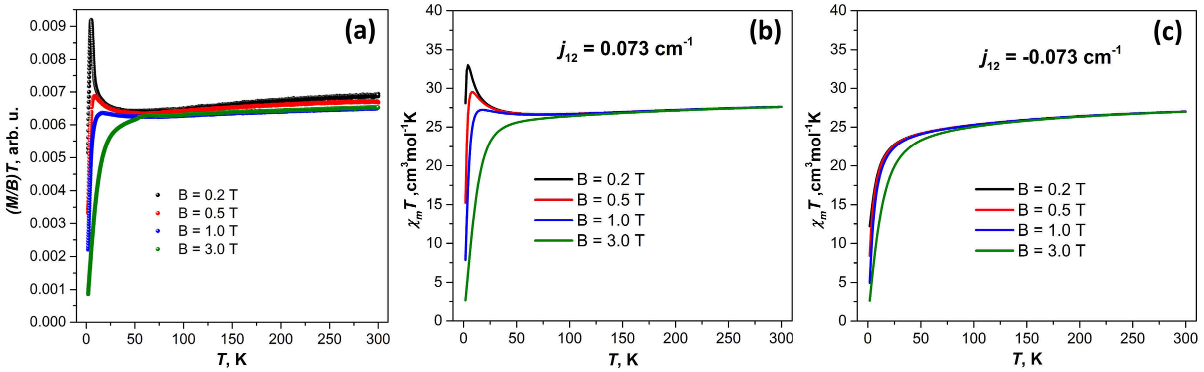

Figure S11. (a) Experimentally measured *(M/B)T* curves (identical to *χT* curves when measured in low field); (b) simulated *χT* curves for the ferromagnetically coupled system; (c) simulated *χT* curves for the antiferromagnetically coupled system.

The ferromagnetic coupling can be also confirmed by the shape of the magnetization curve measured at 8 K (the lowest temperature at which hysteresis is negligible). The simulated magnetization curves are compared to the experimental one at Fig. S12. Whereas the FM-coupled system provides a very good match to the experimental data, the simulated curve for the AFM system deviates significantly.

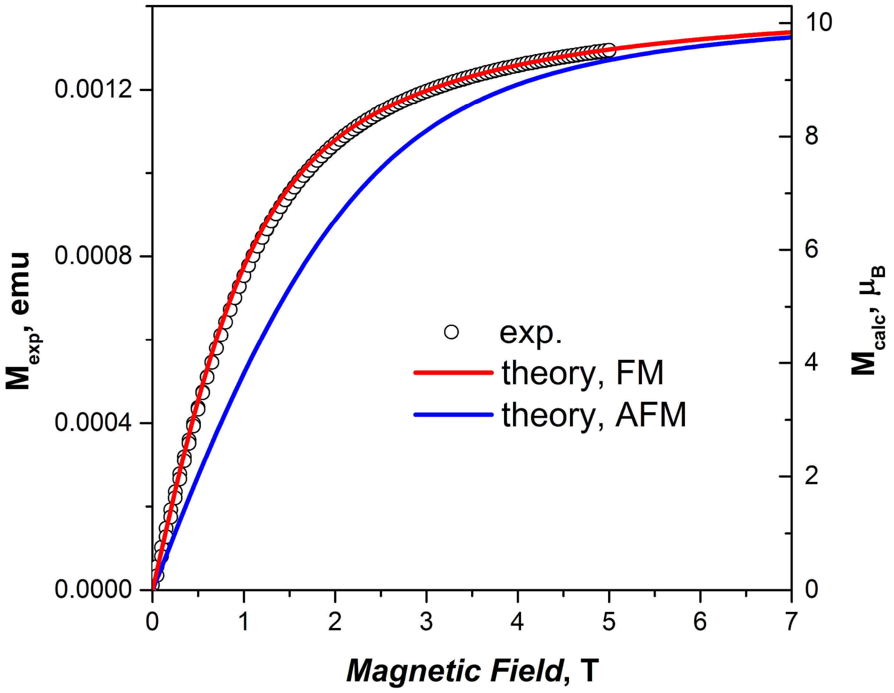

Figure S12. Experimentally measured magnetization curve of $Dy_2ScN@C_{80}$ (dots) compared to the simulations for FM (j_{12} = +0.073 cm^{-1}) and AFM (j_{12} = −0.073 cm^{-1}) coupled system. T = 8 K.

Dipolar contribution to the Dy-Dy interaction energy can be calculated straightforwardly using the equation:

$$E^{dip}(\vec{\mu}_1,\vec{\mu}_2) = -\frac{\mu_0}{4\pi R_{12}^3}\left(3(\vec{n}_r\cdot\vec{\mu}_1)(\vec{n}_r\cdot\vec{\mu}_2)-(\vec{\mu}_1\cdot\vec{\mu}_2)\right) \quad \text{(S2)}$$

where $\vec{n}_r$ is the normal of the radius vector connecting two magnetic moments $\vec{\mu}_1$ and $\vec{\mu}_2$, and R_{12} is the distance between them. The angle between the moments, 116.7°, is taken from *ab initio* calculations. ΔE^{dip} = 4.6 K is somewhat less than a half of the low-temperature barrier in $Dy_2ScN@C_{80}$. From ΔE^{dip}, the dipolar contribution j_{12}^{dip} to the j_{12} constant in eq. (S1) is computed by scaling with the factor of $15^2\cdot\cos(\alpha)$, where α is the angle between the anisotropy axes of individual Dy ions. For $Dy_2ScN@C_{80}$, this amounts to 0.031 cm^{-1}.

Low-temperature magnetization curves of $Dy_2ScN@C_{80}$ also show a feature near 1 T, which can be seen below 3-4 K, but is less clear at higher temperatures. Detailed exploration of this feature in experimental curves is hardly possible because of the slow relaxation of magnetization and hence hysteresis observed at the temperatures when this feature is present. Computed thermodynamic magnetization curves also show the presence of the bent in low-T curves (Fig. S13). Figure S14, illustrating Zeeman splitting of the lowest-energy states in $Dy_2ScN@C_{80}$, shows that may be cause be the level crossing of FM and AFM states in a finite field. Since the measurements are performed for the powder sample with random orientations of the Dy_2ScN cluster with respect to the external field, position of the level crossing is distributed in a rather large field range.

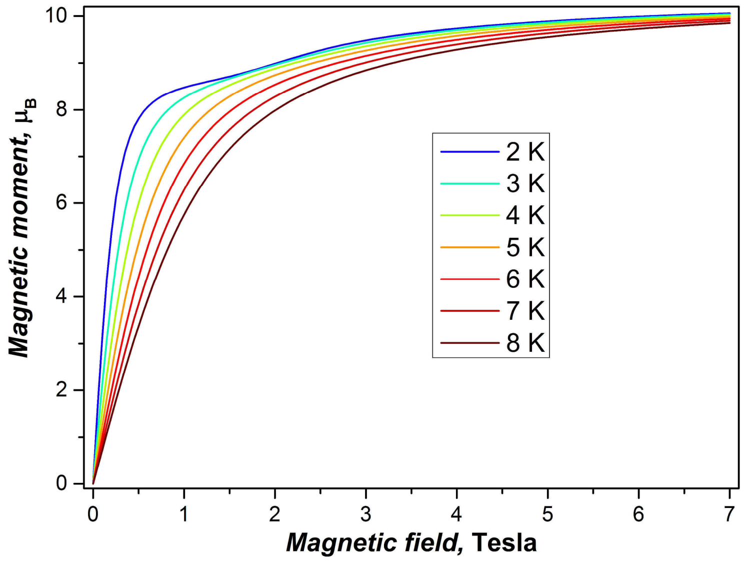

Figure S13. Simulated low-temperature magnetization curves of the $Dy_2ScN@C_{80}$ powder.

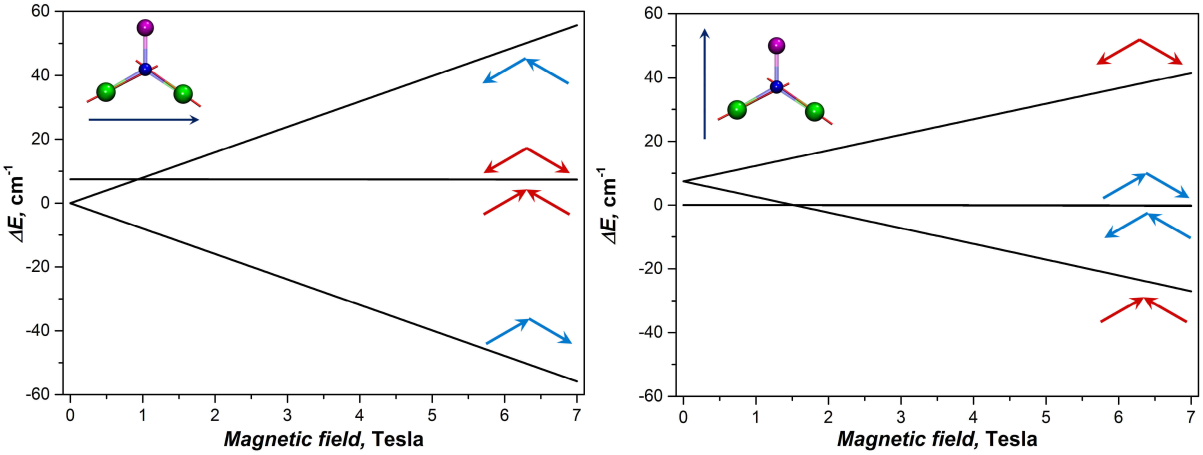

Figure S14. Zeeman splitting of the FM and AFM levels in $Dy_2ScN@C_{80}$ molecule with two different orientations of the external field with respect to the Dy_2ScN cluster. Red and blue arrows show arrangement of the magnetic moments of individual Dy centers (red – AFM coupling, blue – FM coupling).

Excited states of the di-Dy systems in the low-energy part of the spectrum are additive of the values of the constituent single-ions. For instance, 4 states (2 quasi-dublets) with J_z = ± 15/2 for both Dy ions in FM and AFM arrangement, $|\pm15/2, \pm15/2\rangle$, are followed by 8 $|\pm15/2, \pm13/2\rangle$ and $|\pm13/2, \pm15/2\rangle$ states in which one of the Dy centers has J_z = ± 13/2 (the energies of the quasi-dublets are 418, 425, 460, and 467 cm^{-1}; compare to the single-ion KDs with the energies of 418 and 457 cm^{-1} for Dy2 and Dy1). The next 8 states (4 quasi-dublets) with the energies of 746, 752, 758, and 764 cm^{-1} correspond to $|\pm15/2, \pm11/2\rangle$ and $|\pm11/2, \pm15/2\rangle$ states (corresponding single-ion state have the energies of 741 and 755 cm^{-1}). Two $|\pm13/2, \pm13/2\rangle$ quasi-dublets have the energies of 878 and 885 cm^{-1} (compare to the sum of the energy of single-ion state, 418+457 = 875 cm^{-1}). The density of states is then increasing dramatically at higher energies as more and more mixed states become available in the middle part of the spectrum (Fig. S15). As the model describing coupling of Dy moments (Hamiltonian S1) is most probably oversimplified, we doubt that these mixed states are described reliably. Besides, in weakly coupled system, such as $Dy_2ScN@C_{80}$, relaxation via excited states of the coupled system would require simultaneous flip of the spin of two Dy centers which appears less probable than relaxation via excited states of individual Dy ions.

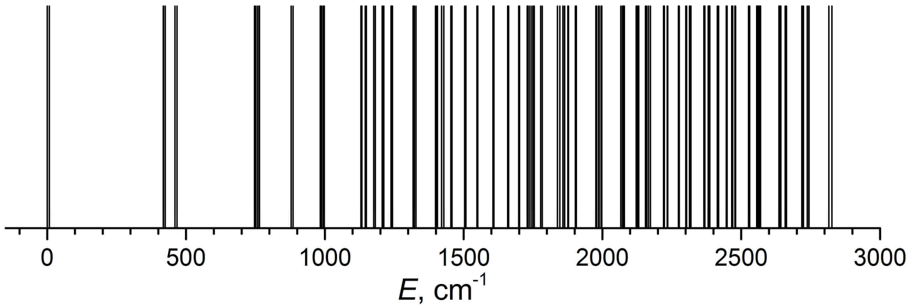

Figure S15. Energy spectrum of the total spin Hamiltonian for $Dy_2ScN@C_{80}$. Although Dy_2ScN is not a Kramers system, each vertical line in the spectrum corresponds to *de facto* to two quasi degenerate states.

S7. References

1. S. Yang, L. Zhang, W. Zhang and L. Dunsch, *Chem.-Eur. J.*, 2010, **16**, 12398-12405.
2. T. Wei, F. Liu, S. Wang, X. Zhu, A. A. Popov and S. Yang, *Chem. Eur. J.*, 2015, **21**, 5750-5759.
3. U. Mueller, R. Förster, M. Hellmig, F. U. Huschmann, A. Kastner, P. Malecki, S. Pühringer, M. Röwer, K. Sparta, M. Steffien, M. Ühlein, P. Wilk and M. S. Weiss, *Eur. Phys. J. Plus*, 2015, **130**, 141.
4. K. M. Sparta, M. Krug, U. Heinemann, U. Mueller and M. S. Weiss, *J. Appl. Crystallogr.*, 2016, **49**, 1085-1092.
5. G. Sheldrick, *Acta Cryst. C*, 2015, **71**, 3-8.
6. A. A. Popov and L. Dunsch, *J. Am. Chem. Soc.*, 2008, **130**, 17726-17742.
7. S. Yang, A. A. Popov and L. Dunsch, *Angew. Chem.-Int. Edit. Engl.*, 2008, **47**, 8196-8200.

S8. DFT-optimized Cartesian coordinates of $Y_2ScN@C_{80}$ conformers

***C*$_3$-derived $Y_2ScN@C_{80}$** (corresponds to the experimental Dy_2ScN site with occupancy 0.69)

Y	8.848857270	2.668527070	2.695707480
Y	8.364416110	4.362144020	5.825169260
Sc	5.857413290	4.388329370	3.307719730
N	7.600977500	3.838249710	3.932342980
C	4.987102560	6.147706390	5.824618350
C	4.902340700	4.978723520	6.653506640
C	4.172543760	3.974288490	5.925829030
C	3.764529180	4.527446850	4.649438480
C	4.281877060	5.887075470	4.583893450
C	4.694682920	6.482606660	3.337097510
C	4.478906280	5.707204990	2.113761100
C	3.937766030	4.322021030	2.172546810
C	3.622582770	3.705735550	3.467278870
C	3.956313890	2.317818930	3.652398670
C	4.624286640	1.522801270	2.637500420
C	5.083169070	2.135146560	1.445564950
C	4.742708030	3.526498030	1.246959360
C	5.699291870	4.379405340	0.596036820
C	6.939758870	3.889070530	0.046121130
C	7.209580470	2.489591650	0.153366090
C	6.298369520	1.638647210	0.863140720
C	7.065657900	0.597697040	1.491894280
C	8.467385170	0.753110300	1.127945410
C	8.556707390	1.955905740	0.287655620
C	9.691880650	2.857373720	0.360553070
C	10.790018760	2.474390680	1.246480180
C	10.709915290	1.257110700	2.093724010
C	9.518328980	0.400771420	2.071486560
C	9.056886180	-0.106737830	3.343190550
C	9.717503950	0.175419670	4.606201760
C	10.796525550	1.093958650	4.656678020
C	11.257663770	1.626404380	3.396216200
C	11.716244160	2.988183450	3.350462570
C	11.806988920	3.818149460	4.525418240
C	11.443615300	3.240747720	5.777453540
C	10.933916030	1.901095350	5.835561010
C	9.971694400	1.841843450	6.901732140
C	9.904503650	3.142137320	7.549336310
C	10.823450620	4.018781020	6.831293450
C	10.550747950	5.423404060	6.662388970
C	9.367272330	5.966825040	7.327057480
C	8.433837940	5.089251650	8.081552730

C	8.683758980	3.639700280	8.149983820
C	7.548946470	2.757973940	8.003655110
C	6.198647720	3.237987320	7.768855580
C	5.953915310	4.623390590	7.564040300
C	7.079946290	5.524965970	7.718995150
C	7.171472230	6.656810880	6.827461730
C	8.560656030	6.912167000	6.562902960
C	8.951044050	7.460858310	5.280116500
C	7.924574870	7.847402210	4.365188400
C	6.536188910	7.609505350	4.655792520
C	6.141063580	6.981735760	5.866008710
C	5.850304100	7.333706700	3.410740150
C	6.837681010	7.404973910	2.354924520
C	8.109566740	7.718795480	2.942146360
C	9.318445030	7.181281490	2.401077410
C	9.227865850	6.402277150	1.205134740
C	7.950515190	6.083092340	0.625448760
C	6.739463280	6.547643170	1.223899670
C	5.571550550	5.698825370	1.148000600
C	8.047276030	4.770567690	0.041275150
C	9.390205160	4.264599240	0.250078540
C	10.109953510	5.289968630	0.977837100
C	11.099053990	4.930058730	1.933733080
C	11.397493990	3.522753840	2.058396360
C	11.603152940	5.211748660	4.367498530
C	11.251242600	5.762760300	3.085863650
C	10.374799910	6.880650930	3.313293290
C	10.188501420	7.020148880	4.733694680
C	10.949885270	5.988431770	5.400106420
C	4.476622640	2.588942690	6.082997760
C	4.328468970	1.760426320	4.933437010
C	5.208004410	0.645651820	4.705789990
C	6.263569850	0.340146420	5.618739940
C	6.356406110	1.124589850	6.813055670
C	5.468505210	2.229044860	7.048343810
C	7.631127560	1.443861110	7.394629010
C	8.839300820	0.978262760	6.801694470
C	8.747822010	0.110458290	5.665396150
C	7.474130690	-0.197611630	5.076764210
C	7.658661350	-0.333320490	3.656854210
C	6.636721880	0.053965800	2.746469790
C	5.386171810	0.493349680	3.286265240

***C_s*-derived $Y_2ScN@C_{80}$** (corresponds to the experimental Dy_2ScN site with occupancy 0.31)

N	7.736609670	3.741768590	4.225521930
C	4.996388470	6.142920840	5.817083960
C	4.901626410	4.975450330	6.664050260
C	4.146154120	3.981228260	5.935600460
C	3.787613110	4.530086940	4.658607820
C	4.309382050	5.878680170	4.571221420
C	4.671037330	6.510984190	3.320981760
C	4.507105210	5.720388230	2.104075970
C	4.116371160	4.321669700	2.247393240
C	3.735496040	3.707284080	3.496989900
C	3.959821400	2.309972010	3.659213940
C	4.622627490	1.532285210	2.643415300
C	5.090859710	2.141729970	1.448721710
C	4.809065190	3.541234790	1.264543360
C	5.680657190	4.416181010	0.529851730
C	6.923790500	3.889850400	0.019983290
C	7.182606870	2.487827910	0.143007150
C	6.278294510	1.622195240	0.848066420
C	7.046411950	0.596359560	1.495883330
C	8.451092670	0.809377930	1.197670130
C	8.518781560	1.997113760	0.362935460
C	9.631358260	2.879328360	0.451695790
C	10.724926490	2.478779570	1.319070010
C	10.745236920	1.225677740	2.084771560
C	9.525171180	0.397517920	2.072590420
C	9.076996880	-0.154273220	3.338187430
C	9.748929700	0.152080170	4.595378840
C	10.879598220	1.037333090	4.647067110
C	11.398993770	1.547693960	3.380024630
C	11.778478170	2.953852320	3.348556460
C	11.836855100	3.806678010	4.519278140
C	11.465893560	3.225862900	5.767946560
C	10.969044070	1.872927710	5.819691230
C	9.990709390	1.821935130	6.880392280
C	9.879999510	3.135182800	7.482731840
C	10.802624870	3.998262160	6.781934460
C	10.507143220	5.378504600	6.576693060
C	9.305141320	5.879579100	7.171104620
C	8.399984700	5.021479620	7.909280080
C	8.671592140	3.616289020	8.108398890
C	7.520336450	2.708694010	8.171940670
C	6.142024410	3.219938020	7.935793620
C	5.909866130	4.636185940	7.639431560

C	7.047351890	5.519941290	7.681820090
C	7.147783320	6.676780390	6.812752670
C	8.534231240	6.895460870	6.514351100
C	8.941674010	7.433982390	5.253144090
C	7.909905320	7.809047940	4.328049970
C	6.520263510	7.613895180	4.622173220
C	6.133108810	6.991576390	5.856879000
C	5.800038860	7.422774460	3.371466700
C	6.788961630	7.488292000	2.283402390
C	8.087117560	7.702965560	2.908401510
C	9.316613070	7.174673830	2.383583690
C	9.219700700	6.396411840	1.196721980
C	7.923499010	6.090512370	0.618265180
C	6.679518360	6.646317560	1.100025340
C	5.496592020	5.785639840	1.003143360
C	8.038780660	4.766461800	0.033898490
C	9.376873410	4.275075090	0.244832090
C	10.102470920	5.285806460	0.965958340
C	11.087928620	4.917009080	1.930931970
C	11.385743360	3.510722540	2.083584570
C	11.615896930	5.202311320	4.349024980
C	11.249739780	5.753750050	3.067777110
C	10.372712320	6.874665720	3.294893250
C	10.186410090	7.004462900	4.716420150
C	10.963674100	5.983804770	5.370466080
C	4.465507430	2.599997190	6.082173180
C	4.323092580	1.760208570	4.943255300
C	5.209366450	0.645582860	4.710177780
C	6.273201150	0.360027860	5.603802720
C	6.378666630	1.175009240	6.785113270
C	5.477717820	2.258909500	7.061189590
C	7.634980280	1.459109160	7.421550160
C	8.853649610	0.981471520	6.798128590
C	8.755669050	0.129464380	5.655144370
C	7.484256580	-0.179963990	5.067331920
C	7.667710250	-0.333406470	3.652096600
C	6.629289930	0.055128190	2.747539340
C	5.382664720	0.493115390	3.289229520
Y	9.150523660	2.448685330	3.346753970
Y	6.685321090	5.101353030	3.001410510
Sc	7.415832190	3.593419490	6.124707050
